# Evaluation and mitigation of potential errors in radiochromic film dosimetry due to film curvature at scanning

**DOI:** 10.1120/jacmp.v16i2.5141

**Published:** 2015-03-08

**Authors:** Antony L. Palmer, David A. Bradley, Andrew Nisbet

**Affiliations:** ^1^ Medical Physics Department Portsmouth Hospitals NHS Trust Portsmouth Hampshire UK; ^2^ Department of Physics Faculty of Engineering and Physical Science, University of Surrey Guildford Hampshire UK

**Keywords:** film dosimetry, Gafchromic EBT3, film curvature, quality control, uncertainty

## Abstract

This work considers a previously overlooked uncertainty present in film dosimetry which results from moderate curvature of films during the scanning process. Small film samples are particularly susceptible to film curling which may be undetected or deemed insignificant. In this study, we consider test cases with controlled induced curvature of film and with film raised horizontally above the scanner plate. We also evaluate the difference in scans of a film irradiated with a typical brachytherapy dose distribution with the film naturally curved and with the film held flat on the scanner. Typical naturally occurring curvature of film at scanning, giving rise to a maximum height 1 to 2 mm above the scan plane, may introduce dose errors of 1% to 4%, and considerably reduce gamma evaluation passing rates when comparing film‐measured doses with treatment planning system‐calculated dose distributions, a common application of film dosimetry in radiotherapy. The use of a triple‐channel dosimetry algorithm appeared to mitigate the error due to film curvature compared to conventional single‐channel film dosimetry. The change in pixel value and calibrated reported dose with film curling or height above the scanner plate may be due to variations in illumination characteristics, optical disturbances, or a Callier‐type effect. There is a clear requirement for physically flat films at scanning to avoid the introduction of a substantial error source in film dosimetry. Particularly for small film samples, a compression glass plate above the film is recommended to ensure flat‐film scanning. This effect has been overlooked to date in the literature.

PACS numbers: 87.55.Qr, 87.56.bg, 87.55.km

## I. INTRODUCTION

Radiochromic film dosimetry is an important tool in radiotherapy to validate absolute dose or dose distributions in challenging external beam or brachytherapy situations.^1^, ^2^, ^3^, ^4^ The use of modern radiochromic films and advanced scanning techniques have seen improved accuracy of film dosimetry and widespread adoption of the technique.^5^, ^6^ Film dosimetry has several advantages over other dosimetry methods including very high spatial resolution in continuous 2D, low‐energy dependence, and near water equivalence. However, care must be taken to reduce uncertainties associated with film dosimetry including film batch and scan orientation consistency, postexposure time, scanner warm‐up characteristics, and lateral film position on the scanner.^(7)^ There are published recommendations for accurate film dosimetry, notably AAPM Task Group 55^(8)^ and much work published in the literature to reduce uncertainties in specific aspects of film dosimetry techniques.^9^, ^10^, ^11^, ^12^, ^13^ However, no prior publication has considered the uncertainty introduced in film dosimetry by film curling at the point of scanning, which is often undetected or deemed insignificant by the operator, but may introduce a substantial dose error. Two commonly used flatbed scanners for film dosimetry, the Epson 10000XL and the Epson V750, both have a gap of several millimeters between the scanner glass plate and scanner lid which permits natural curling of film at scanning.

**Figure 1:**
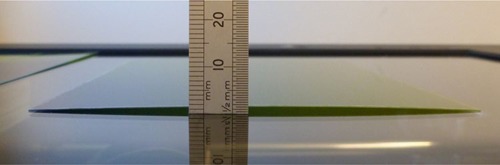
Natural curvature of a 10×10 cm Gafchromic EBT3 film placed on a flatbed scanner plate. In this example, there is a maximum height of 1.5 mm from the glass plate along the film central axis.

Due to the manufacture process, film naturally curls (Fig. 1), particularly for smaller sample sizes (in which the weight of the film cannot suppress the tendency to curl), and the extent of curling and the implications on scanner response and derived dose have not previously been reported. Physically small film samples are often used in the verification of external beam small field dosimetry, or in brachytherapy applications, when small scales are required. As an example, Hassani et al.^(4)^ used film pieces of 3 cm×3 cm and 5×20 cm, analyzed with a flatbed scanner, which is typical practice. Such film sizes are, however, susceptible to curling. There are also phantoms which intentionally hold film in curved positions for irradiation, which may increase the extent of any natural curvature at subsequent scanning.

The mechanism by which deviations from perfectly flat film at scanning result in variations in scanner response is not clear, and the magnitude of the effect has not been reported to date. In an email communication, the flatbed scanner manufacturer Epson has reported that “any [film] curve will cause a change in the resultant scan due to the media potentially being outside the focal plane of the scan head. Additionally it can affect the uniformity of lighting. We would recommend the film is held flat in a frame during scanning. A fluid mount kit may be an option.” (e‐Service Support Technician at Epson Customer Inter@ction Centre, personal communication, email received January 7,2014.)

There is no information in the literature on film curvature or height above scan plane at scanning for dosimetry applications, but there has been discussion of film height above imaging plane in photographic film scanning and optical photographic developers and enlargers. In photographic applications, small changes in film position can have marked changes in the resulting signal due to the Callier effect^(14)^ which describes changes in optical density resulting from variations in illumination, such as from a collimated to diffuse source within the optical system. Any curved film will also have different effective film thicknesses for light paths at different scan positions and will affect surface scattering from the film.

In the present work, we evaluate the effect of film curling at scanning for film dosimetry in experimental tests and a clinical radiotherapy case, and quantify the significance in the context of other film dosimetry uncertainties. We propose and evaluate mitigation methods for film curling including the use of triple‐channel dosimetry analysis methods and a simple compression glass plate at scanning.

## II. MATERIALS AND METHODS

All measurements were performed with Gafchromic EBT3 film (Ashland ISP Advanced Materials, NJ) from a single batch (Lot #A05151203). Films were scanned in red‐green‐blue (rgb) format using a 48‐bit (16 bit per channel) scanner (Epson Perfection V750 Pro; Hemel Hempstead, Hertfordshire, UK) at 72 dpi, in transmission mode, with no color or sharpness corrections and consistent orientation and time‐since‐exposure protocol. The procedure summary recommendations for handling radiochromic film as defined by Niroomand‐Rad et al.^(8)^ in AAPM TG‐55 were adopted. Images were converted to dose profiles or dose maps using FilmQAPro software (Ashland ISP Advanced Materials, version 2.0.4631) using single‐channel dosimetry and rgb triple‐channel dosimetry algorithm.^(9)^ Films were calibrated in a conventional manner with exposures over the range 0 to 20 Gy, from a nominal 6 MV linear accelerator, traceably calibrated to a primary standard at the National Physical Laboratory (Teddington, UK), using the average film pixel value in 4×4 cm regions centered on the beam axis. All profiles across films were measured in a direction aligned with the scan direction to avoid any lateral scanner effect due to polarization.

Three situations were investigated:

1. Test Cast A. EBT3 test film of size 20×20 cm, irradiated with a 6 MV linear accelerator, at 5 cm deep in Solid Water (RMI457, Gammex, WI, USA), with a 20×20 cm field to 7.5 Gy. The film was scanned with controlled curvature, held in contact with the scanner plate at two opposite edges (using tape) and raised at the center by 1, 2, and 4 mm above the scanner plate (using spacers at edges of the film only, with the film held in tension). The film was also scanned perfectly flat at 0 mm height, achieved by taping the edges of the film to the scanner plate, and then achieved with a compression glass plate of 2 mm thickness above the film. Finally, the film was scanned horizontally flat raised uniformly 1 mm from the scanner plate. The film was held under tension to prevent any curvature at 1 mm height. Dose profiles using single red‐channel and triple‐channel dosimetry were compared for the flat and raised film scans.
2. Test Case B. EBT3 test film irradiated with the experimental setup as above, with three 3×3 cm fields to doses of 1.8, 3.5, and 6 Gy, respectively. The film was scanned horizontally flat with 5 mm vertical displacement above the scanner plate, and flat with no vertical displacement, the latter with and without a glass compression plate. The scans were taken with the film horizontally flat, at height above the scanner, to evaluate whether the change in pixel value is a result of film curvature only or height of film above the conventional scan plane. The 5 mm height represents an extreme case of film positioning difference from the conventional position in contact with the scanner. This 5 mm is the maximum gap between scanner glass and scanner lid for the Epson V750 scanner model. The three dose level squares were aligned along the scan direction axis of the scanner to prevent any effects of lateral scanner uncertainty. Dose profiles were separately created using single‐channel red and green dosimetry (the colors most commonly used for film dosimetry) and compared for the flat and raised film scans.
3. Test Case C. EBT3 film of a clinical dose distribution was scanned flat and with natural film curvature. A 10×10 cm EBT3 film was previously irradiated with a typical clinical cervix brachytherapy dose distribution planned with the Nucletron Oncentra Brachy (version 4.1.0.132) treatment planning system using a Nucletron CT/MR treatment applicator, delivered with the Nucletron microSelectron treatment unit (Nucletron, Veenendaal, The Netherlands) using an Ir‐192 high‐dose‐rate brachytherapy source. The resulting film dose range was 2 to 13 Gy. The film was positioned in a liquid water tank for irradiation using the BRAD audit phantom, as described by Palmer et al.^(15)^ The film was scanned flat under a glass compression plate and also scanned allowing the natural curve in the film to be exhibited, which gave a maximum 1.5 mm height at the center of the film while in contact with the scanner plate at two opposite edges, as shown in Fig. 1. Dose maps were calculated for the flat and naturally curved cases. Gamma evaluation index analysis^(16)^ was calculated using FilmQAPro software comparing the treatment planning system intended dose distribution and the film‐measured dose distributions, for the flat and naturally curved film scans.

A glass compression plate has been used to achieve flat film scan conditions in the above methodology. EBT3 film may be used in contact with the glass scanner plate and the glass compression plate film without the formation of light reflection interference patterns, such as Newton's rings, since the film is formed of matte polyester layers with silica particles at the surface creating a 5 μm air gap to the glass plates, which is significantly larger than the light wavelength. Other films, such as the predecessor EBT2, may suffer from interference artifact when in contact with either the upper or lower glass plates, since they are constructed with smooth polyester layers.

## III. RESULTS & DISCUSSION

**Figure 2:**
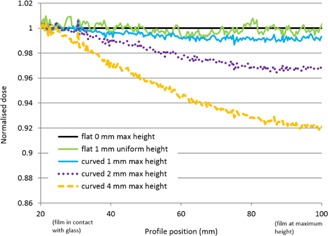
Normalized dose profiles using single red‐channel dosimetry across 6 MV linac beam, from edge of field in contact with the scanner glass (shown at 20 mm abscissa scale) to central axis of field at variable height above scanner glass (shown at 100 mm abscissa scale). (Profile direction is physically aligned with scan direction to avoid any lateral scanner effect due to polarization.)

**Figure 3:**
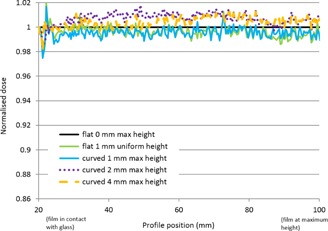
Normalized dose profiles using triple‐channel dosimetry (from FilmQAPro software) across 6 MV linac beam, from edge of field in contact with the scanner glass (shown at 20 mm abscissa scale) to central axis of field at variable height above scanner glass (shown at 100 mm abscissa scale). (Profile direction is physically aligned with scan direction to avoid any lateral scanner effect due to polarization.)

For Test Case (A), 2, 3 show the effect on dose profiles of controlled curvature of EBT3 film irradiated by a 6 MV linear accelerator to approximately 750 cGy. Profiles across half of the film are shown in the figures, corresponding to the range from edge to center of the linear accelerator field. The film is in contact with the scanner plate to the left of the graph, with upward curvature and maximum height achieved at the right of the graph, corresponding to the center of the film. Dose profiles were generated with single red‐channel dosimetry in Fig. 2, and with triple‐channel dosimetry in Fig. 3. In Fig. 2, there is a clear dose reduction (increase in scanned optical density pixel value) with curvature of the film above the scanner plate, 1% reduction at 1 mm height, 3.5% reduction at 2 mm and 8% reduction at 4 mm height. For the equivalent case with triple‐channel dosimetry dose calibration, Fig. 3, there are no systematic changes to the dose profile with increasing height above scanner, and the triple‐channel algorithm effectively corrects the effect. There was no difference in profiles between film held flat with tape or under a glass compression plate in either single‐channel or triple‐channel dosimetry. The above investigation considers the consistency between film scans in the two situations. It is assumed that the flat‐film case is closer to the true value and is in better agreement with the treatment planning system calculation. This is confirmed in Case (C) below.

**Figure 4:**
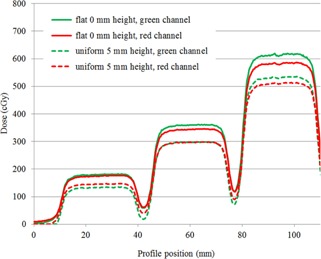
Dose profiles using red and green single‐channel dosimetry across three dose levels, 1.8, 3.5, and 6 Gy, for EBT3 film flat in contact with the scanner glass plate, and with the film flat and raised uniformly 5 mm from the scanner plate. (Profile direction is physically aligned with scan direction to avoid any lateral scanner effect due to polarization.)

For Test Case (B), Fig. 4 shows profiles across three 3×3 cm fields from a linear accelerator at dose levels of 1.8, 3.5, and 6 Gy, for EBT3 film held flat and raised uniformly by 5 mm above the scanner glass plate. The single‐channel dosimetry analysis profiles in red and green channels are represented in Fig. 4. There is a clear reduction in reported dose with the film raised 5 mm compared to the case in contact with the scanner plate. The average dose reduction in the red channel at 1.8 Gy was 21%, at 3.5 Gy was 17%, and at 6 Gy was 14%. The average dose reduction in the green channel at 1.8 Gy was 33%, at 3.5 Gy was 21%, and at 6 Gy was 16%.

**Figure 5:**
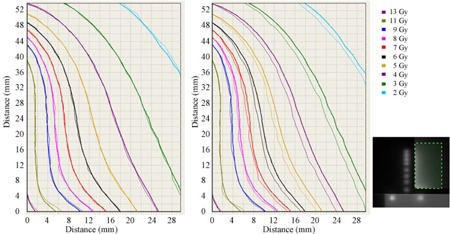
Isodose distributions of film‐measured dose (thin lines) and treatment planning system‐calculated dose (thick lines) over a range 2 to 13 Gy, in a plane anterio–lateral to a cervix treatment applicator brachytherapy dose distribution (shown inset), shown left with film flat on scanner plate and right with film naturally curled on scanner plate (in contact with the plate at 0 mm abscissa scale, rising to a maximum 1.5 mm above the scanner plate at maximum of the abscissa scale).

For Test Case (C), Fig. 5 provides isodose overlay comparisons of film‐measured and treatment planning system‐calculated dose distributions for a typical cervix brachytherapy treatment, with the film scanned flat under a glass compression plate (Fig. 5, left) and with the film permitted to exhibit its natural curvature (Fig. 5, right). When scanned flat there was good agreement between film‐measured and calculated isodoses. With natural film curvature, there was good agreement where the film was in contact with the glass plate but poorer agreement where the film was displaced above the scanner, leading to a maximum 2 mm shift in isodose line for a 1.5 mm vertical height offset, for the dose gradient considered in this example. Gamma criteria passing rates for the two scan situations are provided in Table 1. Gamma was calculated over a film region 3×5 cm (shown inset in Fig. 5 with respect to the source dwell positions), with no lower dose cut‐off. At 3% (local normalization) and 2 mm criteria, there is a 4.3% reduction in passing rate when comparing the curved to the flat scanned film. At the tighter criteria of 2% (local normalization) and 1 mm, there is a dramatic 66.1% reduction in passing rate for the two film scans

**Table 1:** Gamma evaluation passing rate comparing film‐measured dose and treatment planning system‐calculated dose for a typical cervix brachytherapy treatment distribution, with film scanned flat under a glass compression plate and with film allowed to naturally curve on the scanner up to a maximum height of 1.5 mm above scan plane

	*Gamma Passing Rate at:*	
	*3% (local normalization) and 2 mm*	*2% (local normalization) and 1 mm*
Film scanned flat under glass compression plate	100.0%	94.2%
Film scanned with natural curvature	95.7%	28.1%

## IV. CONCLUSIONS

At the currently achieved uncertainty levels for film dosimetry (quoted between 1% and 10% depending on the situation),^17^, ^18^, ^19^ we have demonstrated that film curling during scanning can be a substantial error source if not addressed. This confirms initial results by Palmer et al.^(20)^ who demonstrated apparent shifts in isodose positions due to film curling, but did not fully evaluate the effect. Displacement of film above the scanner plate by 1 to 2 mm can increase pixel values and reduce reported dose by 1% to 4%. From the data presented, the physical height of the film above the scanner plate appeared to cause changes in scanned pixel value and, hence, reported dose, irrespective of any inherent curvature of the film. Increasing the height of the film above the scanner plate increased the effect on scanned pixel value. We found no difference with films scanned flat using adhesive tape on the scanner or scanned under a compression glass plate, with the latter being more convenient.

We have demonstrated that moderate natural film curling, as may be experienced with small film pieces, may be a significant source of uncertainty and error in film dosimetry. A glass compression plate may be conveniently used to flatten small film pieces, and triple‐channel dosimetry is able to mitigate the detrimental effect on film dosimetry accuracy when scanning curved films. The magnitude of the effect is likely to be dependent on the film type and the scanner model used, as well as scanning protocols, and results presented here should be confirmed for local film dosimetry situations. Film curvature must be considered and controlled alongside the other uncertainties present in film dosimetry.

## ACKNOWLEDGMENTS

The authors gratefully acknowledge Ashland ISP, USA, and Vertec, UK, for the supply of Gafchromic EBT3 film used in this investigation.
